# Polymorphisms in Protamine 1 and Protamine 2 predict the risk of male infertility: a meta-analysis

**DOI:** 10.1038/srep15300

**Published:** 2015-10-16

**Authors:** Weijun Jiang, Hui Sun, Jing Zhang, Qing Zhou, Qiuyue Wu, Tianfu Li, Cui Zhang, Weiwei Li, Mingchao Zhang, Xinyi Xia

**Affiliations:** 1 Department of Reproduction and Genetics, Institute of Laboratory Medicine, Jinling Hospital, Nanjing University School of Medicine, Nanjing 210002, P.R. China; 2 Department of Diagnostic Ultrasound, Jinling Hospital, Nanjing University School of Medicine, Nanjing 210002, P.R. China

## Abstract

Several studies have investigated the association between polymorphisms in protamine 1 and 2 genes and male infertility risk, with inconsistent results to date. This meta-analysis based on the 13 published case-control studies, including 7350 cases and 6167 controls, was performed to further establish the potential association between the 6 common single nucleotide polymorphisms (rs35576928, rs737008, rs35262993, rs2301365, rs1646022, rs2070923) in protamines 1 and 2 and male infertility. The -190C > A (rs2301365) polymorphism was identified as a risk factor for male infertility under all models. Interestingly, rs1646022 and rs737008 polymorphisms exerted protective effects against male sterility in Asian and population-based under some models. No associations between the remaining SNPs and male sterility were observed.

Infertility is a widespread reproductive health problem affecting family well-being and social stability worldwide. Globally, around 15% of heterosexual couples are unable to conceive children without assistance^1^. Couples at higher risk for infertility are increasing in number, and the majority of cases are idiopathic^2^. Male infertility contributes to half of these cases^3^. Although several factors can lead to male infertility, such as malformations of the reproductive tract (cryptorchidism or varicocele, orchitis, karyotype anomalies, hypogonadotrophic hypogonadism and Y chromosome microdeletions), infection, and chemical exposure^3^,^4^,^5^,^6^,^7^, 50–70% of male infertility is of unknown etiology, much of which is possibly genetic. To determine the underlying causes, extensive research on the genetic causes of male infertility has been performed in recent years^4^.

Protamines, which were first isolated from spermatozoa a century ago, play vital roles in spermatogenesis. The nucleoprotein gene products, protamine 1 (PRM1, NC_000016.9, GI: 224589807) and protamine 2 (PRM2, NC_000016.9, GI: 224589807), are closely linked in a stretch of DNA 13–15 kb long on human chromosome 16p13.3, along with the gene encoding transition protein 2 (TNP2), categorized as members of the protamine gene family. Protamine is the major DNA-binding protein in sperm nucleus that promotes DNA condensation and packaging in spermatozoa by histone replacement during spermatogenesis. The structure of chromatin undergoes constant remodeling involving complex morphologic, physiologic and biochemical modifications^8^,^9^. Mutations or polymorphisms within protamine genes induce conformational changes of the encoded proteins and alter their incorporation into sperm chromatin, leading to sperm defects. Deficiency of PRM1 and PRM2 in mice results in sperm morphology defects, motility reduction and infertility due to haploinsufficiency^10^,^11^. Consistently, clinical studies in humans have demonstrated an association of PRM1 and PRM2 variants with male infertility. A number of case-control genetic studies have been conducted, but the majority of patients had a clinical phenotype of slightly defective spermatogenesis. Mutations in protamine genes are reported to cause abnormal spermatogenesis and defects in imprinting and induce sperm chromatin damage and DNA breaks, although the underlying mechanisms remain largely unknown^12^.

A number of molecular epidemiological studies have been conducted to examine the association between PRM1 and PRM2 polymorphisms and male infertility in diverse populations. Among these, rs2301365 of PRM1 is in the 5′- untranslated regions (5′-UTR), rs2070923 and rs1646022 of PRM2 are located in the intron, while rs35262993 and rs737008 are two synonymous single nucleotide polymorphisms (SNPs) involving nucleotide exchange at positions 54 (exon 1) and 230 (exon 2). In addition, a non-synonymous SNP at position 102 (exon 1) resulting in the amino acid substitution Arg34Ser (A34R) has been extensively investigated^12^,^13^,^14^,^15^,^16^,^17^,^18^,^19^,^20^,^21^,^22^. However, data from these studies are inconsistent or even contradictory. The majority of studies to date have analyzed the above polymorphisms in small sample sizes, leading to underestimation of the association. There was no meta-analysis of exploring the exactly association between the SNPs and male infertility reported so far. To ascertain the effects of the polymorphisms (rs35576928, rs737008, rs35262993, rs2301365, rs1646022, rs2070923) on the risk of male infertility and quantify potential between-study heterogeneity, we conducted a meta-analysis of 13 eligible and published case-control studies.

## Results

### Study characteristics

**Figure 1:**
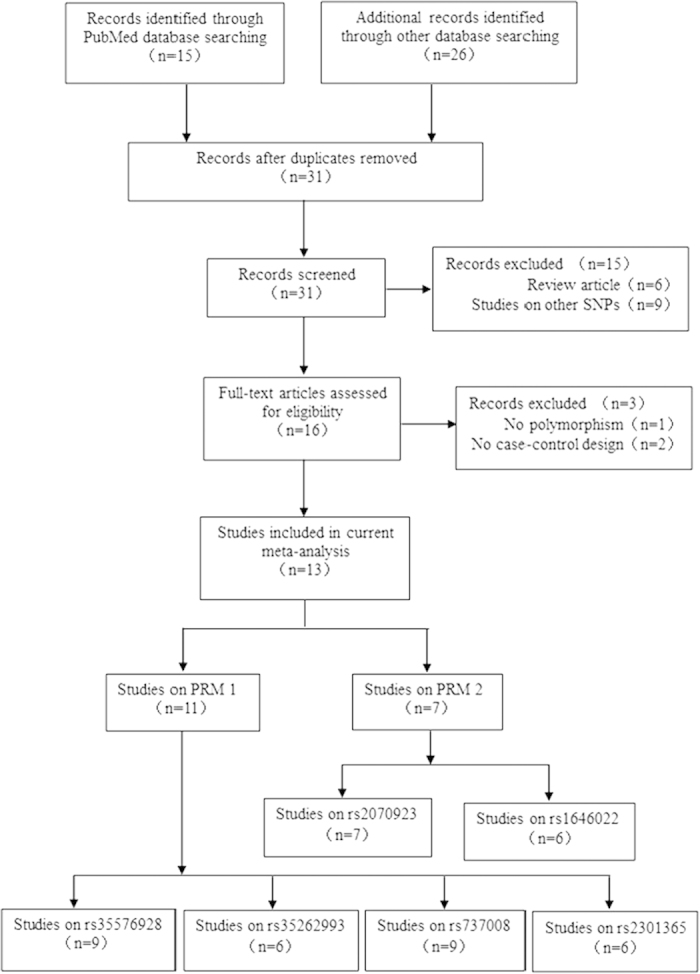
Flow diagram of the study selection process.

Through literature search and selection based on inclusion criteria, 13 articles were identified after reviewing potentially relevant articles (Fig. 1). The characteristics of the selected studies are presented in Tables 1, 2, 3 and Supplementary Tables S1–S3. Publication dates range from 2003 to 2012.

In total, 9 studies on the rs737008 polymorphism, including 1447 cases and 1284 controls, met the inclusion criteria and were selected for meta-analysis. The number of cases included varied from 53 to 304, with a mean (±standard deviation, SD) of 160.78 (±70.26) and controls varied from 50 to 376, with a mean (±SD) of 142.67 (±104.11). The characteristics of all nine studies are summarized in Table 1. Specific data on the other SNPs are presented in Tables 2, 3 and Supplementary Tables S1–S3.

### Meta-analysis of the rs737008 polymorphism and male infertility

**Figure 2:**
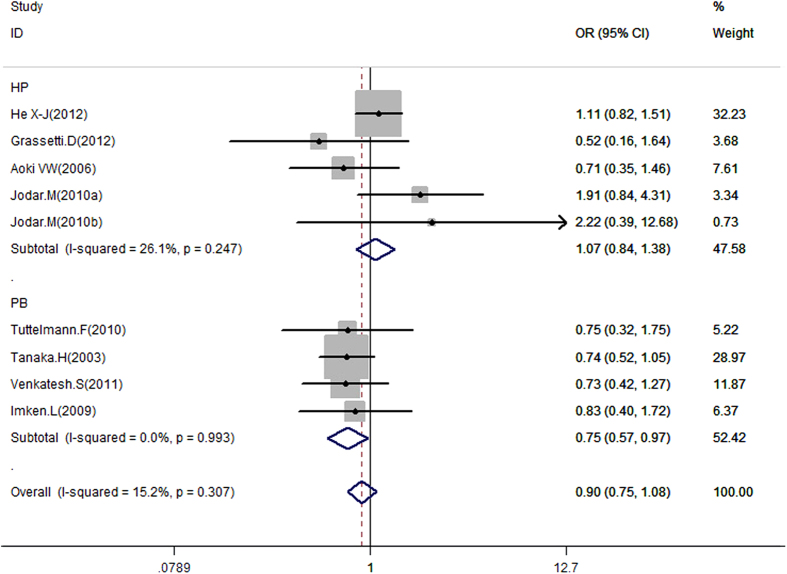
Forest plot of the rs737008 polymorphism and male infertility risk in the dominant model. Studies were plotted according to the last name of the first author (followed by the publication year in parentheses). Horizontal lines represent 95% CI. Each square represents the OR point estimate, and its size is proportional to the weight of the study. The diamond (and broken line) represent the overall summary estimate, with confidence interval given by its width. The unbroken vertical line is at the null value (OR = 1.0). CI, confidence interval; OR, odds ratio.

Data on the association between rs737008 and male infertility risk are summarized in Table 4, Fig. 2 and Supplementary Figure S1. In overall analysis, no significant association was observed between rs737008 and male infertility under the four genotype models. To clarify differences in potential ethnicity and control sources, subgroup analysis was conducted. Significant association was found between the rs737008 polymorphism and male infertility in subgroups of population based under the dominant model (for AA + CA *vs*. CC: OR = 0.75, 95% CI = 0.57–0.97, *P* = 0.030), while no such correlation was observed for the other subgroups, such as the Asian group, under the heterozygous model (for CA *vs*. CC: OR = 0.88, 95% CI =  = 0.70–1.11, *P* = 0.269) and the Caucasian group under the dominant model (for CA + AA *vs*. CC: OR = 0.91, 95% CI = 0.64–1.29, *P* = 0.592). Specific data are presented in Table 4.

### Meta-analysis of the rs2301365 polymorphism and male infertility

**Figure 3:**
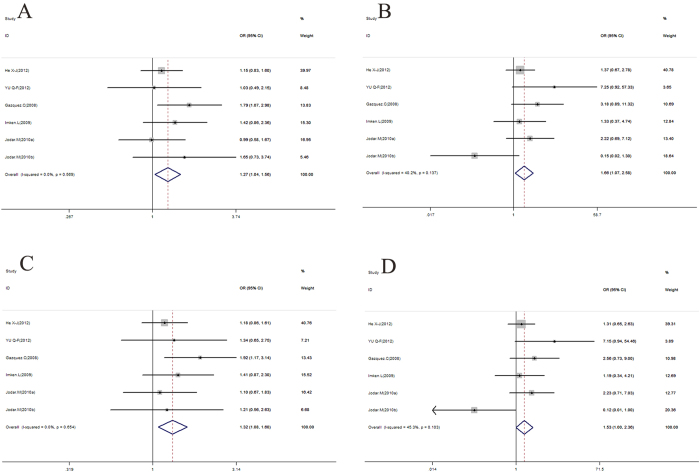
Forest plot for the association between rs2301365 and male infertility for fixed effects. (**A**) Heterozygous model. (**B**) Homozygous model. (**C**) Dominant model. (**D**) Recessive model. For each study, the point estimate of OR (the size of the square is proportional to the weight of each study) and 95% CI for OR (extending lines) is shown. Pooled OR and 95% CI are presented as diamonds.

Data on the association between the rs2301365 polymorphism and male infertility risk are summarized in Table 5, Fig. 3 and Supplementary Figures S2–S3. Significant associations were observed between rs2301365 and an elevated risk of male infertility under all models in overall analysis (for AA + CA *vs*. CC: OR = 1.32, 95% CI = 1.08–1.60, *P* = 0.006; for CA *vs*. CC: OR = 1.27, 95% CI = 1.04–1.56, *P* = 0.022; for AA *vs*. CC: OR = 1.66, 95% CI = 1.07–2.58, *P* = 0.024; for AA + CA *vs*. CC: OR = 1.53, 95% CI = 1.00–2.36, *P* = 0.052; for A *vs*. C: OR = 1.28, 95% CI = 1.09–1.51, *P* = 0.003). Subgroup analyses revealed significant associations within the Caucasian subgroup (for CA *vs*. CC: OR = 1.40, 95% CI = 1.06–1.85, *P* = 0.015; for CA + AA *vs*. CC: OR = 1.42, 95% CI = 1.09–1.85, *P* = 0.010; for A *vs*. C: OR = 1.32, 95% CI = 1.06–1.65, *P* = 0.014), the polymerase chain reaction (PCR) sequence subgroup (for CA *vs*. CC: OR = 1.40, 95% CI = 1.06–1.85, *P* = 0.015; for CA + AA *vs*. CC: OR = 1.42, 95% CI = 1.09–1.85, *P* = 0.010; for A *vs*. C: OR = 1.32, 95% CI = 1.06–1.65, *P* = 0.014) and the PB subgroup (for CA + AA *vs*. CC: OR = 1.60, 95% CI = 1.12–2.29, *P* = 0.010; for CA + AA *vs*. CC: OR = 1.65, 95% CI = 1.17–2.33, *P* = 0.005; for A *vs*. C: OR = 1.54, 95% CI = 1.15–2.06, *P* = 0.004), but not the remaining subgroups. Specific data are summarized in Table 5.

### Meta-analysis of the rs1646022 polymorphism and male infertility

**Figure 4:**
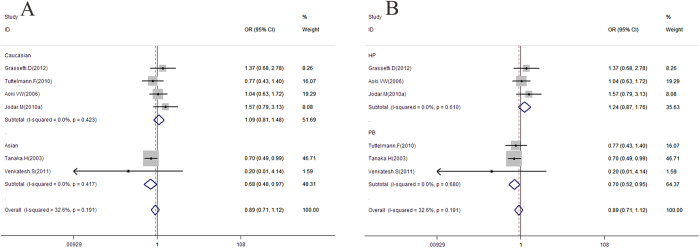
Meta-analysis of male infertility risk associated with rs1646022 with dominant model model for OR. (**A**) Ethnic subgroup analysis. (**B**) Control source subgroup analysis. OR, odds ratio; CI, confidence interval.

Data on the association between rs1646022 and male infertility risk are summarized in Table 6, Fig. 4 and Supplementary Figure S4. Overall, no significant association was evident between rs1646022 and male infertility under all models. To clarify whether potential ethnic and control source differences affect this relationship, subgroup analyses by ethnicity and control sources of study populations were conducted. Significant associations were observed between rs1646022 and estimated risk of male infertility in subgroups of Asians (for CC + GC *vs*. GG: OR = 0.68, 95% CI = 0.48–0.97, *P* = 0.032) and PB (for CC + GC *vs*. GG: OR = 0.70, 95% CI = 0.52–0.95, *P* = 0.023; for C *vs*. G: OR = 0.76, 95% CI = 0.61–0.95, *P* = 0.017). This correlation was not observed in other subgroups under all models. Specific data are described in Table 6.

### Meta-analysis of other SNPs (rs35576928, rs35262993 and rs2070923) and male infertility

No significant association was observed in overall analysis between the SNPs and male infertility under all models. To clarify the effects of potential ethnic, methodological and control source differences, subgroup analyses were performed. No significant association was evident between the polymorphisms examined and estimated risk of male infertility in all subgroups under all models.

### Publication bias and small-study effects

Begg’s funnel plot and Egger’s test were performed to assess publication bias. For all SNPs, funnel plot shapes did not reveal any evidence of obvious asymmetry. Egger’s test was used to provide statistical evidence of funnel plot symmetry. The results suggest no publication bias or small-study effects.

### Sensitivity analysis

We additionally conducted sensitivity analyses on SNPs under all models by omitting one study at a time in the calculation of a summary outcome. Although the sample sizes for cases and controls in all eligible studies varied, corresponding pooled ORs and 95% CIs were not qualitatively altered with or without studies on small samples. No other single study influenced pooled OR and 95% CI qualitatively.

## Discussion

Spermatogenesis is a complex process involving mitotic and meiotic division of germ cells resulting in the formation of haploid spermatozoa. Highly coordinated expression of genes and subtle post-transcriptional regulation are therefore crucial for normal germ cell development. During the process of male reproduction, at least 150 different genes are involved in spermatogenesis. Changes in a cohort of genes and expression patterns affect spermatogenesis and its products, leading to spermatogenesis dysfunction and consequently, male sterility^23^.

To date, two protamine types have been identified in mammals, designated PRM1 and PRM2. The nucleoprotein PRM1 is present in all species of vertebrates while PRM2 exists in some mammalian species, including human and mouse^24^. The following physiological and biological functions of protamine have been identified: (i) paternal genome packing, (ii) competition and removal of transcription factors and other proteins from the spermatid and (iii) imprinting of the paternal genome during spermatogenesis^9^. In addition, accumulating studies indicate that abnormal protamine expression is associated with defective spermatogenesis^25^.

Variants of PRM1 and PRM2 have been shown to be related to male infertility in humans and animals^19^,^22^,^26^. In a mouse model, deficiency of PRM1 and PRM2 results in sperm morphology defects, motility reduction and infertility due to gene haplotype^10^,^11^,^27^. A number of case-control genetic studies have been conducted, but the majority of patients involved had a clinical phenotype of slightly defective spermatogenesis^16^,^17^,^21^. Screening for PRM1 and PRM2 variants by Tanaka *et al*.^20^ in a large cohort of infertile Japanese patients led to the identification of eight novel SNPs (rs187174862, c.160C > A, rs145663132, rs737008, c.431A > G, c.248C > T, rs1646022, rs2070923), none of which caused amino acid changes. Ravel *et al*.^22^ performed direct sequencing analysis for PRM1 in a group of French patients. In this case, SNP rs35576928 (A34R) was detectable only in two patients, one with idiopathic infertility and the other displaying oligozoospermia with increased sperm DNA fragmentation. Tuttelmann and colleagues proposed that 5 SNPs (rs35262993, rs35576928, rs737008, rs1646022 and rs2070923) of PRM1 and PRM2 are associated with mild oligozoospermia (n = 77) or teratozoospermia (n = 88) in a Caucasian population^16^. The group of Aston performed genome-wide analysis of 172 non-obstructive azoospermia (NOA) and severe oligozoospermia patients. None of the three tag SNPs of PRM1 and PRM2 (rs2301365, rs35576928 and rs3177008) were associated with severely defective spermatogenesis.

The present meta-analysis including 7350 cases and 6167 controls from 13 case-control studies explored the association between PRM1 and PRM2 polymorphisms and male infertility. We searched available databases, such as GWAS Central (http://www.gwascentral.org/) and National Human Genome Research Institute GWAS Catalog (http://www.genome.gov/26525384), but failed to identify a relevant genome-wide association (GWAS) study on all SNPs.

Initially, we focused on the relationship between PRM1 and PRM2 polymorphisms and male infertility risk. 6 studies dating from 2006 to 2012 were included. In rs35576928, no mutant homozygotes were identified. We only compared the distribution of GT *vs*. GG (heterozygous model) and T *vs*. G (allele model) among the case and control groups. No association of SNPs of PRM1 with male infertility was observed (OR = 1.20, 95% CI = 0.81–1.77, *P* = 0.358; OR = 1.19, 95% CI = 0.81–1.73, *P* = 0.373) and publication bias (*P* = 0.112; *P* = 0.12) did not exist in the meta-analysis. He *et al*.^13^ showed that the PRM1 variant rs35576928 (R34S) is significantly associated with severe oligozoospermia, even after strict Bonferroni correction, and the dominant model of rs35576928 is a protective factor for spermatogenesis. However, no significance between the cases and controls for this SNP was reported by other investigators. One possible reason for these discrepant findings is specific selection of the clinical subtypes and variations of PRM1 and PRM2 in the different populations tested. Conservation of protamines is of importance in mammals. Minor changes in the coding and non-coding regions of protamine genes may lead to significant abnormalities in their expression or maintenance of gene expression stability. The genetic and molecular mechanisms underlying abnormal protamine expression and their relationship with severely altered spermatogenesis are currently unclear. A number of findings support an association between protamine genes and infertility. Firstly, variants of PRM1 and PRM2 are relatively common in male infertility patients, but rare in fertile men. Secondly, patients with abnormal protamine expression exhibit severe defects in semen quality, including severe oligozoospermia and NOA. Moreover, animal model studies have shown that spermatogenesis is severely altered upon experimental reduction of protamine expression. Therefore, further research on the precise association between the rs35576928 polymorphism and male infertility is warranted.

For rs737008, 9 studies that met the inclusion criteria were selected, including 1447 cases and 1284 controls dating from 2003 to 2012. In overall analysis, no significant association was observed between the polymorphism and male infertility under all models. However, a strongly protective effect was specifically observed between rs737008 and male infertility in the PB subgroup under the dominant model (for CA + AA *vs*. CC: OR = 0.75, 95% CI = 0.57–0.97, *P* = 0.03). In addition, an edge effect may exist in the PB subgroup under the heterozygous model (for CA *vs*. CC: OR = 0.75, 95% CI = 0.56–1.00, *P* = 0.051). One of the most important goals of the meta-analysis was to identify the source of heterogeneity. Accordingly, studies were stratified according to ethnicity, control source and method. However, we failed to observe heterogeneity in all subgroups.

The available literature on the relationship between the PRM1 -190C > A (rs2301365) polymorphism and male infertility risk documents controversial findings. We selected six studies dating from 2008 to 2012 that met the inclusion criteria in the meta-analysis, including 1025 cases and 819 controls. The study focused on whether the SNP is a risk for male infertility under all models. Data from our meta-analysis showed that mutation increases the risk of male sterility under all models. A potential explanation for the association between PRM1 -190C > A polymorphism and infertility is that the region encompassing this polymorphism resulted in clear DNase I footprint protection *in vitro*, spanning an adjacent serum response element and the serum extended protection region. In addition, this polymorphism site is located within a potential Eα regulatory element, and binding sites for basonuclin and survival interacting protein 1 are present in the promoter region of the PRM1 gene. Therefore, it is reasonable to hypothesize that the -190C > A polymorphism leads to changes in expression of the PRM1 gene, resulting in abnormal sperm morphology and PRM1/2 content as well as infertility. An abnormal PRM1/2 ratio (involving the appearance of a sperm DNA fragment) has clearly been shown to be associated with male infertility^14^,^17^.

For rs1646022, 6 studies were selected from 2008 to 2012 that met inclusion criteria, including 898 cases and 641 controls. No significant correlation of this SNP, located in the intron area of the PRM2 gene in chromosome 16p13.2, was observed with male sterility in overall analyses under all models. However, a strong protective effect of rs1646022 against male infertility was observed in the Asian subgroup under the dominant model (OR = 0.68, 95% CI = 0.48–0.97, *P* = 0.032) and the PB subgroup under the dominant model (OR = 0.70, 95% CI = 0.52–0.95, *P* = 0.023) and allele model (OR = 0.76, 95% CI = 0.61–0.95, *P* = 0.017). After considering a comprehensive list of factors, the following possible causes were proposed: SNP markedly decreases the risk of male infertility in Asian and PB, since this mutation may lead to some key enzymes failing to cut and joint PRM gene during the protamine translation process. Inconsistent findings with Caucasian and other populations indicate that ethnicity may contribute to differences in male infertility susceptibility. Moreover, progression of male sterility is known to be the outcome of interactions between genes and environment. Caucasians are used to a meat-based diet and bear considerably more stress in terms of lifestyle, which may affect the development of male germ cells. Finally, the joint effects of SNPs on PRM haplotypes should also be considered. An individual with a clinical disorder is not the product of the single disrupted gene. Genetic disruption is embedded within the context of the entire genome of the individual. After applying the nonparametric trim and fill method, we observed no publication bias in SNPs under all models in the meta-analysis.

To the best of our knowledge, this is the largest and most comprehensive meta-analysis performed so far for the quantitative evaluation of the roles of PRM1 rs35262993 and PRM2 rs2070923 in male infertility. Associations between the two SNPs and male infertility were explored in 1182 cases and 871 controls from six case-control studies and 1204 cases and 1018 controls from seven case-control studies, respectively. However, no correlations of the two SNPs with disease were detected. This may be attributable to a number of factors. Firstly, the two SNPs may be non-sensitive sites. We cannot exclude the possibility that these SNPs may be only genetic markers of male infertility in linkage disequilibrium with other mutations or variations that play a role in male infertility. Secondly, studies with a relatively small sample sizes may lack the adequate power to allow accurate conclusions to be drawn. Thirdly, races living in different latitudes with extreme weather are under the influence of the environment, climatic conditions (air temperature, solar radiation, ultraviolet intensity) and varied dietary habits during the lengthy evolution process, which may affect the mode of action and potency of the two SNPs, leading to differences in results among the populations from distinct regions.

A number of limitations of the current meta-analysis need to be addressed. Firstly, only 13 studies were enrolled, and the relatively small total sample size had limited power for exploring the real association. Secondly, subgroup analyses by method, ethnicity and control source involved relatively small groups, which may not impart sufficient statistical power to explore the real association and are more likely to reveal greater beneficial effects than large-scale trials^28^. Thirdly, our results were based on unadjusted estimates. A more precise analysis should be conducted if all data are available, which would allow for adjustment by other co-variants, including body mass index, age, work, smoke or alcohol habits, environmental exposure and other lifestyle factors. In addition, an individual with a clinical disorder is not a result of the single disrupted gene. Genetic disruption is embedded within the context of the individual’s entire genome and environment exposure. Finally, inclusion of zero-event trials can sometimes decrease the effect size estimate and narrow confidence intervals^29^.

In conclusion, data from our meta-analysis support further exploration of the true association between these SNPs and male infertility. The -190C > A (rs2301365) polymorphism is associated with risk for male infertility under all models. Interestingly, the rs1646022 polymorphism has a protective effect against male infertility in Asian and PB populations under dominant and allele models, while the rs737008 polymorphism exerts a protective effect in PB under dominant and heterozygous models. However, no associations were observed between the other SNPs examined and male sterility. In terms of male infertility with multifactorial etiology, further studies with larger sample sizes and stratified by different ethnic backgrounds, environmental exposure or other risk factors are warranted to clarify the potential roles of PRM1 and 2 polymorphisms in the pathogenesis of male infertility.

## Methods

### Study selection

Data from single reports were extracted (Fig. 1). We systematically collected studies published from 2000 to 2014 on PRM1 and PRM2 by searching the common English databases (PubMed and Web of Science) using the following search phrases: PRM1 or Protamine 1, PRM2 or Protamine 2, polymorphism or polymorphisms and male infertility.

Inclusion criteria were as follows: (1) research focus on rs35576928, rs737008, rs35262993, rs2301365, rs1646022, rs2070923 polymorphisms and male infertility, (2) studies on human beings, (3) case-control study design, (4) contained details of genotype frequency of cases and controls, (5) papers where the full text was available. Two reviewers assessed the full text of eligible studies from the above databases. Additional studies were identified from a manual search of references of original or review articles on this topic.

### Data extraction and verification

Information on the enrolled studies is listed in Tables 1, 2, 3 and Supplementary Tables S1-S3, including: (I) the first author’s name, (II) year of publication, (III) ethnicity, (IV) genotyping methods, (V) number of cases and controls, (VI) Hardy-Weinberg equilibrium in the controls, (VII) country or region of origin. Firstly, two reviewers (Weijun Jiang and Cui Zhang) extracted all the data independently, screening the citations that met inclusion criteria. Next, one reviewer extracted while the other cross-checked the data. Disagreements were resolved by review and discussion. Ethnicities were categorized as Asian and Caucasian.

### Statistical analysis

The strength of association between polymorphisms and male infertility risk was assessed based on OR with 95% CI. The combined ORs were respectively calculated for five genetic models (heterozygous, homozygous, dominant, recessive and allele). The statistical significance of pooled OR was determined with the Z-test, with *P*-values < 0.05 considered significant. Heterogeneity across the studies was evaluated with the Chi-square-based Q test^30^, and considered significant at *P* < 0.05. A random-effects model using the DerSimonian and Laird method and fixed-effects model using the Mantel–Haenszel method were used to pool data from the meta-analysis^31^. In cases where the *P*-value for heterogeneity was >0.10 and *I*^*2*^ < 50%, indicating absence of heterogeneity between studies, the fixed-effect model was employed to evaluate the summary OR. Conversely, if the *P*-value for heterogeneity was ≤0.10 or *I*^*2*^ ≥ 50%, indicating a high extent of heterogeneity between studies, the random-effects model was employed to evaluate the summary OR.

The six polymorphisms were evaluated for the associations with male infertility susceptibility based on four genetic models. Compared to the wild-type homozygote (WW), we first estimated the effects of the rare homozygous (RR) and heterozygous (WR) genotypes on risk of infertility, and subsequently evaluated the risk of infertility under four genetic models. In addition, allele model associations were estimated (R *vs*. W). Stratified analysis was further performed based on ethnicity (Asian and Caucasian), method (MassARRAY, PCR sequence, polymerase chain reaction-restriction fragment length polymorphism (PCR-FRLP) and others) and control source (hospital population and population based). A sensitivity analysis was conducted in which a single study within the meta-analysis was deleted each time. To determine the influence of individual data sets on overall pooled OR, forest plot analysis was performed to assess result stability. Funnel plots and Egger’s liner regression test were applied to test publication bias,^32^. All statistical analyses were carried out using STATA version 11.0 (Stata Corporation College Station, TX, USA).

## Additional Information

**How to cite this article**: Jiang, W. *et al*. Polymorphisms in Protamine 1 and Protamine 2 predict the risk of male infertility: a meta-analysis. *Sci. Rep*. **5**, 15300; doi: 10.1038/srep15300 (2015).

## Supplementary Material

Supplementary Information

## Figures and Tables

**Table 1:** Main characteristics of all studies on the genotype of rs737008 included in the meta-analysis

First author (year)	Country	Ethnicity	Method	Control source	Cases/Controls	Case			Control			Case		Control		*P*_*HWE*_	MAF
						CC	CA	AA	CC	CA	AA	C (%)	A (%)	C (%)	A (%)		
He XJ (2012)	China	Asian	MassARRAY	HP	304/376	161	112	31	209	142	25	434 (71.38%)	174 (28.62%)	560 (74.47%)	192 (25.53%)	0.894	0.291
Grassetti D (2012)	Italy	Caucasian	PCR sequence	HP	110/53	15	55	40	4	29	20	85 (38.64%)	135 (61.36%)	37 (34.91%)	69 (65.09%)	0.137	
Tuttelmann F (2010)	Germany	Caucasian	PCR sequence	PB	171/77	23	63	85	8	28	41	109 (31.87%)	233 (68.13%)	44 (28.57%)	110 (71.43%)	0.338	
Aoki VW (2006)	America	Caucasian	PCR sequence	HP	192/96	32	79	81	12	43	41	143 (37.24%)	241 (62.76%)	67 (34.90%)	125 (65.10%)	0.889	
Tanaka H (2003)	Japan	Asian	PCR sequence	PB	226/270	125	86	15	129	117	24	336 (74.34%)	116 (25.66%)	375 (69.44%)	165 (30.56%)	0.729	
Venkatesh S (2011)	India	Asian	PCR sequence	PB	100/100	56	20	24	48	24	28	132 (66%)	68 (34%)	120 (60%)	80 (40%)	0.0001	
Imken L (2009)	Morocco	Caucasian	PCR sequence	PB	135/160	16	55	64	16	74	70	87 (32.22%)	183 (67.78%)	106 (33.13%)	214 (66.88%)	0.579	
Jodar M (2010a)	Spain	Caucasian	PCR sequence	HP	156/102	12	64	80	14	41	47	88 (28.21%)	224 (71.79%)	69 (33.82%)	135 (66.18%)	0.302	
Jodar M (2010b)	Sweden	Caucasian	PCR sequence	HP	53/50	2	28	23	4	20	26	32 (30.19%)	74 (69.81%)	28 (28%)	72 (72%)	0.955	

PCR sequence, polymerase chain reaction and sequencing.

HP, hospital population; PB, population based.

*P*_*HWE*_, Value of Hardy-Weinberg equilibrium in the control group.

MAF, Minimum allele frequency.

**Table 2:** Main characteristics of all studies on the genotype of rs2301365 included in the meta-analysis.

First author (year)	Country	Ethnicity	Method	Control source	Cases/Controls	Case			Control			Case		Control		*P*_*HWE*_	MAF
						CC	CA	AA	CC	CA	AA	C (%)	A (%)	C (%)	A (%)		
He XJ (2012)	China	Asian	MAssARRAY	HP	304/369	187	100	17	241	112	16	474 (77.96%)	134 (22.04%)	594 (80.49%)	144 (19.51%)	0.518	0.222
YU QF (2012)	China	Asian	MAssARRAY	HP	157/37	61	70	26	17	19	1	192 (61.15%)	122 (38.85%)	53 (71.62%)	21 (28.38%)	0.109	
Gazquez C (2008)	Spain	Caucasian	PCR-RFLP and sequence	PB	220/101	114	90	16	68	30	3	318 (72.27%)	122 (27.73%)	166 (82.18%)	36 (17.82%)	0.888	
Imken L (2009)	Morocco	Caucasian	PCR sequence	PB	135/160	85	45	5	113	42	5	215 (79.63%)	55 (20.37%)	268 (83.75%)	52 (16.25%)	0.653	
Jodar M (2010a)	Spain	Caucasian	PCR sequence	HP	156/102	88	55	13	60	38	4	231 (74.04%)	81 (25.96%)	158 (77.45%)	46 (22.55%)	0.501	
Jodar M (2010b)	Sweden	Caucasian	PCR sequence	HP	53/50	25	27	1	26	17	7	77 (72.64)	29 (27.36%)	69 (69%)	31 (31%)	0.147	

PCR sequence, polymerase chain reaction and sequencing; PCR-RFLP, polymerase chain reaction-restriction fragment length polymorphism

HP, hospital population; PB, population based.

*P*_*HWE*_, Value of Hardy-Weinberg equilibrium in the control group.

MAF, Minimum allele frequency.

**Table 3:** Main characteristics of all studies on the genotype of rs1646022 included in the meta-analysis.

First author (year)	Country	Ethnicity	Method	Control source	Cases/Controls	Case			Control			Case		Control		*P*_*HWE*_	MAF
						GG	GC	CC	GG	GC	CC	G (%)	C (%)	G (%)	C (%)		
Grassetti D (2012)	Italy	Caucasian	PCR sequence	HP	110/53	30	62	18	18	26	9	122 (55.45%)	98 (44.55%)	62 (58.49%)	44 (41.51%)	0.94	0.717
Tuttelmann F (2010)	Germany	Caucasian	PCR sequence	PB	159/73	57	66	36	22	28	23	180 (56.60%)	138 (43.40%)	72 (49.32%)	74 (50.68%)	0.047	
Aoki VW (2006)	America	Caucasian	PCR sequence	HP	192/96	76	85	31	39	44	13	242 (63.02%)	142 (36.98%)	124 (64.58%)	68 (35.42%)	0.985	
Tanaka H (2003)	Japan	Asian	PCR sequence	PB	226/269	127	80	19	127	118	24	334 (73.89%)	118 (26.11%)	372 (69.14%)	166 (30.86%)	0.646	
Venkatesh S (2011)	India	Asian	PCR sequence	PB	100/100	100	0	0	98	0	2	200 (100%)	0 (0%)	196 (98%)	4 (2%)	0	
Jodar M (2010a)	Spain	Caucasian	PCR sequence	HP	111/50	35	54	22	21	18	11	124 (55.86%)	98 (44.14%)	60 (60%)	40 (40%)	0.077	

PCR sequence, polymerase chain reaction and sequencing.

HP, hospital population; PB, population based.

*P*_*HWE*_, Value of Hardy-Weinberg equilibrium in the control group.

MAF, Minimum allele frequency.

**Table 4:** Main results on the rs737008 polymorphism in the meta-analysis.

	Cases/Controls	CA *vs.*CC	AA *vs.* CC	AA + CA *vs.* CC	AA *vs.* CA + CC	A *vs.* C	*P*	*P*_*h*_	*I*^*2*^	OR (95% CI)	*P*	*P*_*h*_	*I*^*2*^	OR (95% CI)	*P*	*P*_*h*_	*I*^*2*^	OR (95% CI)	*P*	*P*_*h*_	*I*^*2*^
		OR (95% CI)	*P*	*P*_*h*_	*I*^*2*^	OR (95% CI)															
Total	1447/1284	0.88 (0.73–1.07)	0.206	0.475	0.0%	0.96(0.74–1.25)	0.767	0.259	20.7%	0.90(0.75–1.08)	0.272	0.307	15.2%	1.02(0.85–1.23)	0.836	0.635	0.0%	0.97(0.86–1.09)	0.56	0.341	11.3%
Ethnicity																					
Asian	630/746	0.88(0.70–1.11)	0.269	0.407	0.0%	0.94(0.52–1.69)	0.834	0.079	60.7%	0.90(0.73–1.11)	0.337	0.164	44.7%	1.04(0.73–1.47)	0.831	0.136	49.8%	0.91(0.68–1.22)	0.536	0.059	64.6%
Caucasian	817/538	0.89(0.61–1.29)	0.536	0.328	13.6%	0.94(0.65–1.36)	0.748	0.417	0.0%	0.91(0.64–1.29)	0.592	0.325	14.0%	1.01(0.81–1.27)	0.912	0.833	0.0%	0.99(0.83–1.16)	0.862	0.661	0.0%
Control source																					
HP	815/677	1.01(0.77–1.32)	0.932	0.258	24.6%	1.24(0.86–1.79)	0.239	0.222	30.0%	1.07(0.84–1.38)	0.573	0.247	26.1%	1.11(0.86–1.43)	0.424	0.464	0.0%	1.07(0.91–1.26)	0.39	0.47	0.0%
PB	632/607	0.75(0.56–1.00)	0.051	0.999	0.0%	0.74(0.51–1.07)	0.112	0.931	0.0%	**0.75(0.57**–**0.97)**	**0.03**	**0.993**	**0.0%**	0.92(0.70–1.22)	0.574	0.654	0.0%	0.85(0.72–1.02)	0.074	0.602	0.0%

OR, odds ratio; CI, confidence interval.

*P*_*h*_*, P* value of heterogeneity.

*P* value of Q-test for the heterogeneity test.

*I*^*2*^: 0–25, no heterogeneity; 25–50, modest heterogeneity; 50, high heterogeneity.

Bold font mean statistically significant results.

**Table 5:** Main results on the rs2301365 polymorphism in the meta-analysis.

		CA *vs.* CC	*P*	*P*_*h*_	*I*^*2*^	AA *vs.* CC	*P*	*P*_*h*_	*I*^*2*^	AA + CA*vs.* CC	*P*	*P*_*h*_	*I*^*2*^	AA *vs.* CA + CC	*P*	*P*_*h*_	*I*^*2*^	A *vs.* C	*P*	*P*_*h*_	*I*^*2*^
		OR (95% CI)				OR (95% CI)				OR (95% CI)				OR (95% CI)				OR (95% CI)			
Total	1025/819	**1.27(1.04**–**1.56)**	**0.022**	**0.569**	**0.0%**	**1.66(1.07**–**2.58)**	**0.024**	**0.137**	**40.2%**	**1.32(1.08**–**1.60)**	**0.006**	**0.654**	**0.0%**	**1.53(1.00**–**2.36)**	**0.052**	**0.103**	**45.3%**	**1.28(1.09**–**1.51)**	**0.003**	**0.365**	**8.0%**
Ethnicity																					
Asian	461/406	1.13(0.84–1.53)	0.430	0.783	0.0%	1.85(0.98–3.52)	0.06	0.124	57.8%	1.20(0.90–1.60)	0.212	0.752	0.0%	1.83(0.98–3.44)	0.059	0.106	61.8%	1.24(0.98–1.57)	0.075	0.309	3.4%
Caucasian	564/413	**1.40(1.06**–**1.85)**	**0.017**	**0.434**	**0.0%**	1.50(0.82–2.75)	0.187	0.103	51.5%	**1.42(1.09**–**1.85)**	**0.01**	**0.47**	**0.0%**	1.23(0.43–3.49)	0.698	0.078	56.0%	**1.32(1.06**–**1.65)**	**0.014**	**0.233**	**29.8%**
Method																					
MassARRAY	461/406	1.13(0.84–1.53)	0.430	0.783	0.0%	1.85(0.98–3.52)	0.06	0.124	57.8%	1.20(0.90–1.60)	0.212	0.752	0.0%	1.83(0.98–3.44)	0.059	0.106	61.8%	1.24(0.98–1.57)	0.075	0.309	3.4%
PCR squence	564/413	**1.40(1.06**–**1.85)**	**0.017**	**0.434**	**0.0%**	1.50(0.82–2.75)	0.187	0.103	51.5%	**1.42(1.09**–**1.85)**	**0.01**	**0.47**	**0.0%**	1.23(0.43–3.49)	0.698	0.078	56.0%	**1.32(1.06**–**1.65)**	**0.014**	**0.233**	**29.8%**
Control source																					
HP	670/558	1.14(0.88–1.46)	0.318	0.764	0.0%	1.47(0.51–4.21)	0.472	0.069	57.7%	1.18(0.93–1.50)	0.173	0.98	0.0%	1.38(0.45–4.24)	0.573	0.04	63.8%	1.18(0.97–1.44)	0.092	0.487	0.0%
PB	355/261	**1.60(1.12**–**2.29)**	**0.01**	**0.534**	**0.0%**	2.17(0.91–5.19)	0.081	0.337	0.0%	**1.65(1.17**–**2.33)**	**0.005**	**0.391**	**0.0%**	1.83(0.77–4.34)	0.172	0.397	0.0%	**1.54(1.15**–**2.06)**	**0.004**	**0.329**	**0.0%**

OR, odds ratio; CI, confidence interval.

*P*_*h*_*, P* value of heterogeneity.

*P* value of Q-test for the heterogeneity test.

*I*^*2*^: 0–25, no heterogeneity; 25–50, modest heterogeneity; 50, high heterogeneity.

Bold font mean statistically significant results.

**Table 6:** Main results on the rs1646022 polymorphism in the meta-analysis.

	Cases/Controls	GC*vs.* GG	*P*	*P*_*h*_	*I*^*2*^	CC*vs.* GG	*P*	*P*_*h*_	*I*^*2*^	CC + GC*vs.* GG	*P*	*P*_*h*_	*I*^*2*^	CC *vs.* GC + GG	*P*	*P*_*h*_	*I*^*2*^	C *vs.* G	*P*	*P*_*h*_	*I*^*2*^
		OR (95% CI)				OR (95% CI)				OR (95% CI)				OR (95% CI)				OR (95% CI)			
Total	898/641	0.93(0.73–1.19)	0.556	0.188	33.1%	0.89(0.63–1.25)	0.495	0.602	0.0%	0.89(0.71–1.12)	0.339	0.191	32.6%	0.87(0.64–1.19)	0.389	0.709	0.0%	0.91(0.77–1.07)	0.252	0.232	27.0%
Ethnicity																					
Asian	326/369	0.69(0.48–1.01)	0.055	0.374	0.0%	0.73(0.39–1.36)	0.321	0.378	0.0%	**0.68(0.48**–**0.97)**	**0.032**	**0.417**	**0.0%**	0.86(0.47–1.57)	0.615	0.323	0.0%	0.77(0.58–1.01)	0.057	0.185	43.2%
Caucasian	572/272	1.16(0.84–1.61)	0.364	0.485	0.0%	0.97(0.64–1.45)	0.87	0.489	0.0%	1.09(0.81–1.48)	0.557	0.423	0.0%	0.88(0.61–1.26)	0.482	0.579	0.0%	1.00(0.81–1.23)	0.994	0.381	2.2%
Control source																					
HP	413/199	1.26(0.87–1.83)	0.229	0.418	0.0%	1.21(0.73–2.00)	0.454	0.999	0.0%	1.24(0.87–1.76)	0.229	0.61	0.0%	1.04(0.66–1.63)	0.873	0.809	0.0%	1.12(0.88–1.43)	0.36	0.954	0.0%
PB	485/442	0.74(0.54–1.02)	0.07	0.524	0.0%	0.67(0.42–1.08)	0.099	0.620	0.0%	**0.70(0.52**–**0.95)**	**0.023**	**0.680**	**0.0%**	0.74(0.48–1.15)	0.178	0.473	0.0%	**0.76(0.61**–**0.95)**	**0.017**	**0.41**	**0.0%**

OR, odds ratio; CI, confidence interval.

*P*_*h*_*, P* value of heterogeneity.

*P* value of Q-test for heterogeneity test.

*I*^*2*^: 0–25, no heterogeneity; 25–50, modest heterogeneity; 50, high heterogeneity.

Bold font mean statistically significant results.
