# Supplementary material for: Polymorphisms in Protamine 1 and Protamine 2 predict the risk of male infertility: a meta-analysis
Source: Sci Rep. 2015 Oct 16;5:15300. doi: 10.1038/srep15300 (PMC4607923; doi:10.1038/srep15300)
Supplement: Supplementary Information [file srep15300-s1.pdf]

Polymorphisms in Protamine 1 and Protamine 2 predict the risk of male infertility: a meta-analysis

Weijun Jiang[1, +], Hui Sun[2, +], Jing Zhang[1], Qing Zhou[1], Qiuyue Wu[1], Tianfu Li[1], Cui Zhang[1], Weiwei Li[1], Mingchao Zhang[1] and Xinyi Xia[1, *]

[1]Department of Reproduction and Genetics, Institute of Laboratory Medicine, Jinling Hospital, Nanjing University School of Medicine, Nanjing 210002, P.R. China

[2]Department of Diagnostic Ultrasound, Jinling Hospital, Nanjing University School of Medicine, Nanjing 210002, P.R. China

Email addresses:

WJJ: 1048281756@qq.com

[+]HS: fmmusunhui@sina.com

JZ: 710081649@qq.com

QZ: 863840726@qq.com

QYW: 475626760@qq.com

TFL: talentli13@163.com

CZ: 841479480@qq.com

WWL: eeeeet@sina.com

MCZ: zmchj99@163.com

*XYX: xiaxynju@163.com

* Correspondence Author

[+] The authors contributed equally to this work

Figure legends:

Supplementary Figure S1. Meta-analysis of the rs737008 polymorphism and male infertility risk. (A) Heterozygous model. **(B)** Homozygous model. **(C)** Dominant model. **(D)** Recessive model. OR, odds ratio; CI, confidence interval.

Supplementary Figure S2. Meta-analysis of the rs2301365 polymorphism and male infertility risk. (A) Ethnic subgroup analysis in heterozygous model. **(B)** Ethnic subgroup analysis in dominant model. **(C)** Control source subgroup analysis in heterozygous model. **(D)** Control source subgroup analysis in dominant model. OR, odds ratio; CI, confidence interval.

Supplementary Figure S3. Meta-analysis of the rs2301365 polymorphism and male infertility risk in allele model. (A) Overall group analysis. **(B)** Ethnic subgroup analysis. **(C)** Method subgroup analysis. **(D)** Control source subgroup analysis. OR, odds ratio; CI, confidence interval.

Supplementary Figure S4. Meta-analysis of the rs1646022 polymorphism and male infertility risk under allele model in control source subgroup analysis. OR, odds ratio; CI, confidence interval.

Supplementary Figure S1

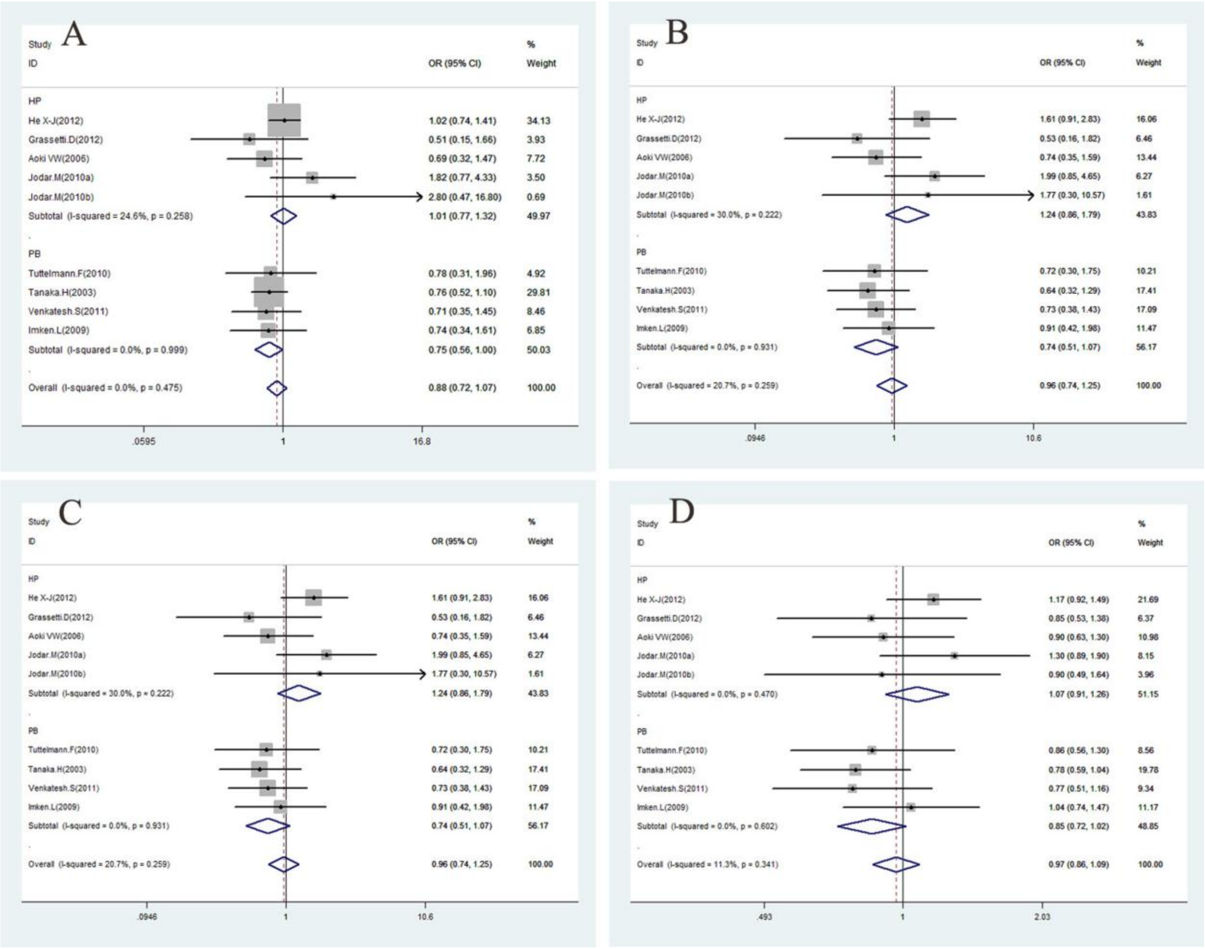

Supplementary Figure S2

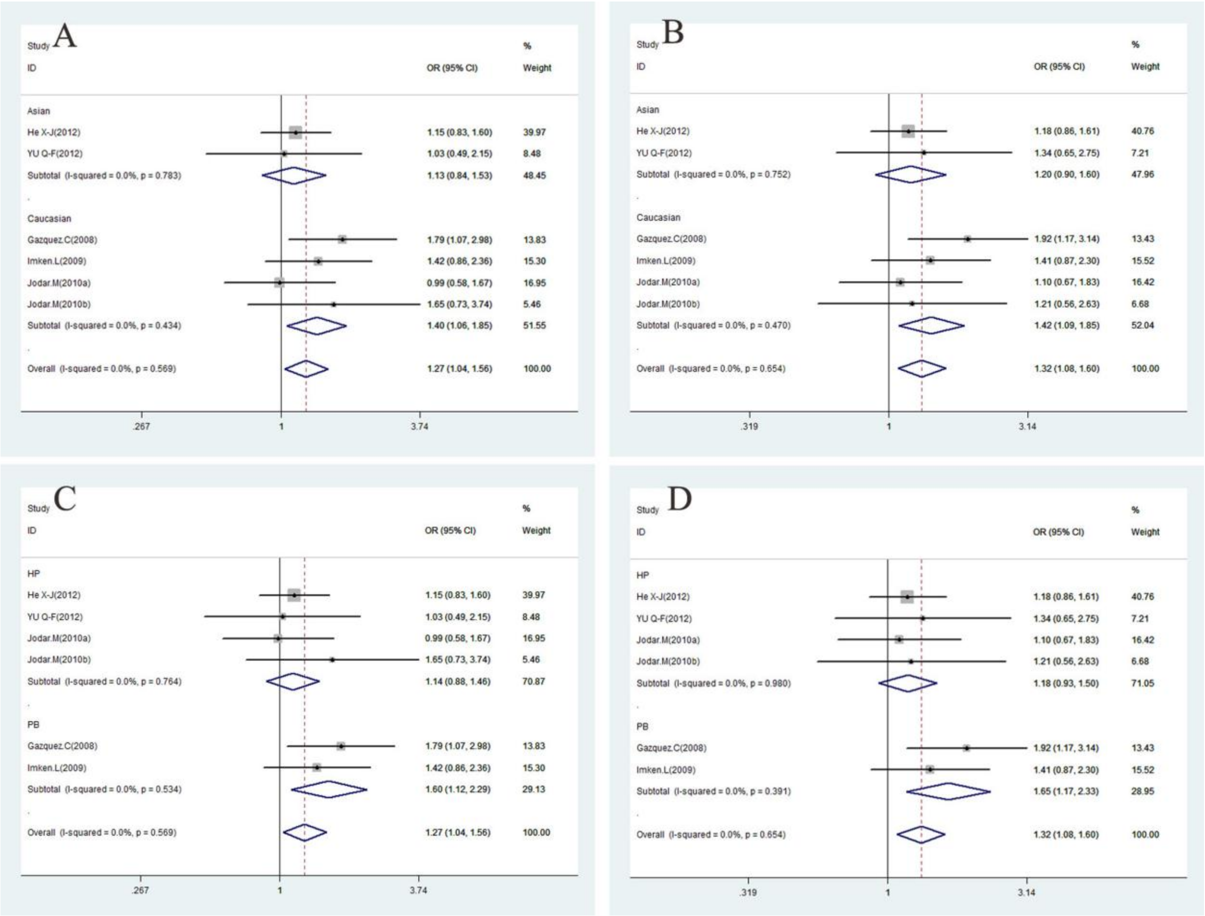

Supplementary Figure S3

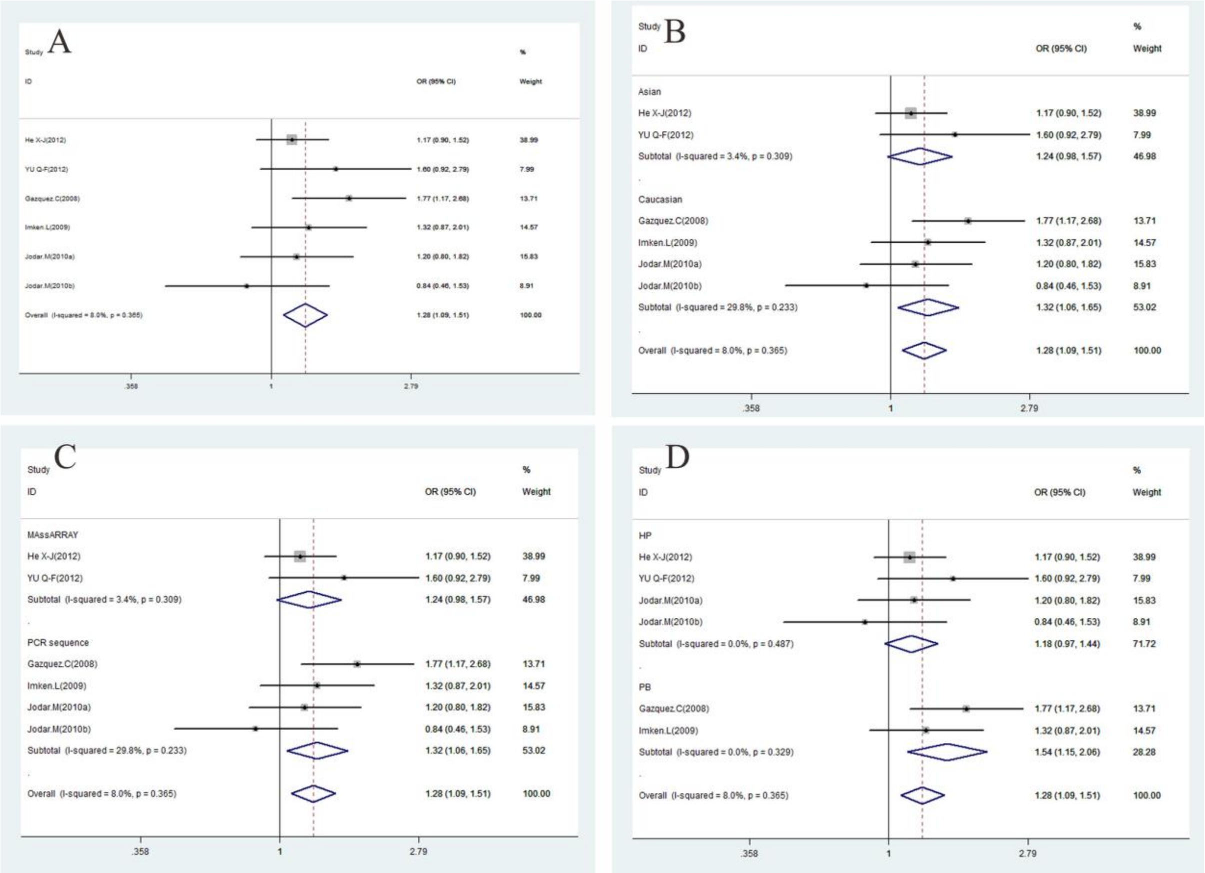

Supplementary Figure S4

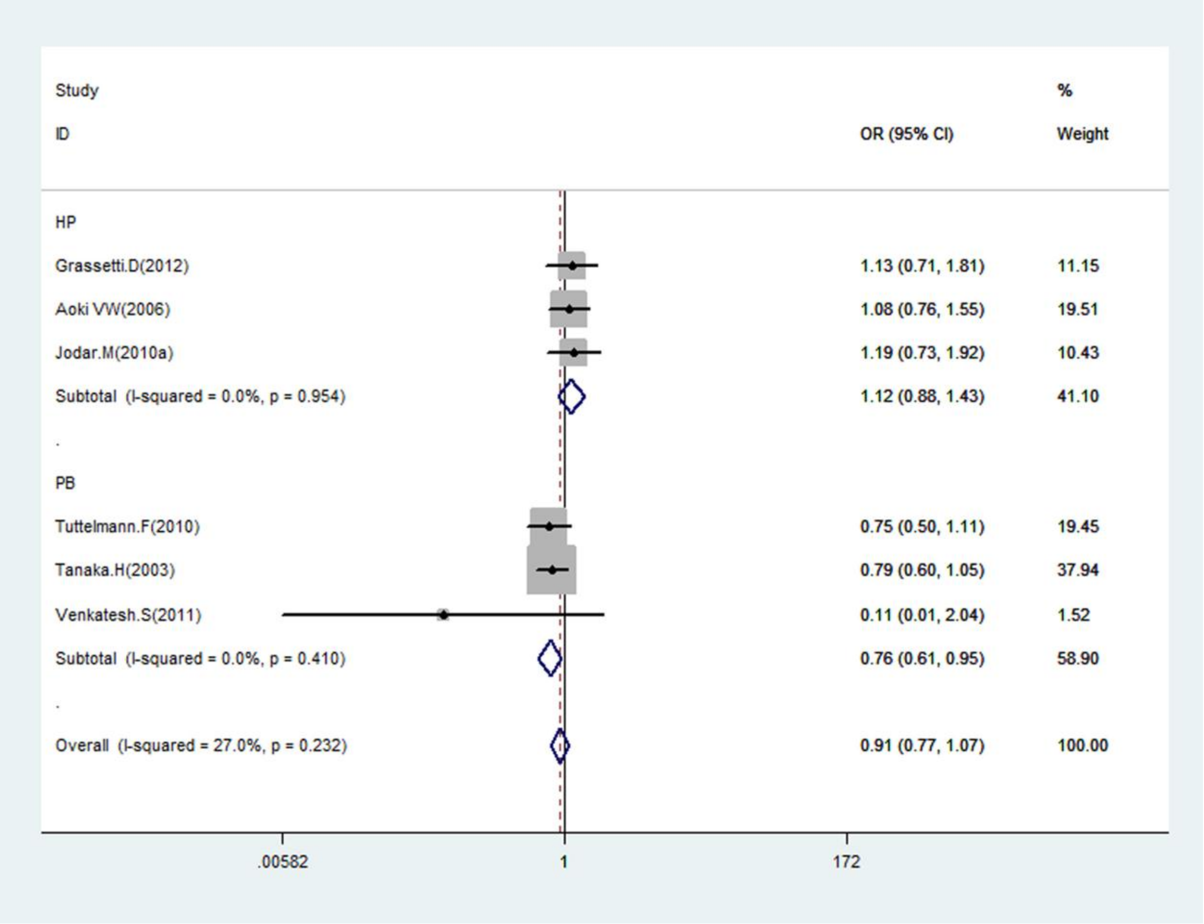

Supplementary Table S1. Main characteristics of all studies on the genotype of rs35576928 included in the meta-analysis

						Case			Control			Case		Control			
First author (year)	Country	Ethnicity	Method	Control source	Cases/ Controls	GG	GT	TT	GG	GT	TT	G (%)	T (%)	G (%)	T (%)	P_{HWE}	MAF
He XJ(2012)	China	Asian	Others	HP	303/373	256	47	0	328	45	0	559 (92.24%)	47 (7.76%)	701 (93.97%)	45 (6.03%)	0.215	0.983
Grassetti D(2012)	Italy	Caucasian	PCR sequence	HP	110/53	110	0	0	52	1	0	220 (100%)	0 (0%)	105 (99.06%)	1 (0.04%)	0.945	
Tuttelmann F (2010)	Germany	Caucasian	PCR sequence	PB	171/77	167	4	0	75	2	0	338 (98.83%)	4 (1.17%)	52 (98.7%)	2 (1.3%)	0.908	
Kichine E(2008)	France	Caucasian	Others	PB	300/672	300	0	0	670	2	0	600 (100%)	0 (0%)	1342 (99.85%)	2 (0.15%)	0.969	
Aoki VW(2006)	America	Caucasian	PCR sequence	HP	192/96	189	3	0	94	2	0	381 (99.22%)	3 (0.78%)	190 (98.96%)	2 (1.04%)	0.918	
Iguchi N(2006)	America	Caucasian	MIX	HP	30/10	27	3	0	9	1	0	57 (95%)	3 (5%)	19 (95%)	1 (5%)	0.868	
Ravel C(2007)	France	MIX	MIX	HP	281/111	269	2	0	111	0	0	561 (99.82%)	1 (0.18%)	222 (100%)	0 (0%)	1	
Jodar M(2010a)	Spain	Caucasian	PCR sequence	HP	156/102	155	1	0	102	0	0	311 (99.68%)	1 (0.32%)	204 (100%)	0 (0%)	1	
Jodar (2010b)	Sweden	Caucasian	PCR sequence	HP	53/50	52	1	0	49	1	0	105 (99.06%)	1 (0.94%)	99 (99%)	1 (1%)	0.943	

HP, hospital population; PB, population based

PCR-RFLP, polymerase chain reaction-restriction fragment length polymorphism; MIX, complete region or PCR sequence and PCR-RFLP; Others, MassARRAY or PCR-RFLP

P_{HWE}, Value of Hardy-Weinberg equilibrium in the control group

MAF, Minimum allele frequency

Supplementary Table S2. Main characteristics of all studies on the genotype of rs35262993 included in the meta-analysis

						Case			Control			Case		Control			
First author (year)	Country	Ethnicity	Method	Control source	Cases/ Controls	GG	GA	AA	GG	GA	AA	G (%)	A (%)	G (%)	A (%)	P_{HWE}	MAF
He XJ(2012)	China	Asian	MAssARRAY	HP	293/374	292	1	0	373	1	0	585 (99.83%)	1 (0.17%)	747 (99.87%)	1 (0.13%)	0.979	0.008
Grassetti D(2012)	Italy	Caucasian	PCR sequence	HP	110/53	109	1	0	53	0	0	219 (99.55%)	1 (0.45%)	106 (100%)	0 (0%)	1	
Tuttelmann F(2010)	Germany	Caucasian	PCR sequence	PB	171/77	167	4	0	75	2	0	338 (98.83%)	4 (1.17%)	152 (98.70%)	2 (1.30%)	0.908	
Aoki VW(2006)	America	Caucasian	PCR sequence	HP	192/96	189	3	0	94	2	0	381 (99.22%)	3 (0.78%)	190 (98.96%)	2 (1.04%)	0.919	
Ravel C(2007)	France	MIX	PCR-RFLP and sequence	HP	281/111	281	0	0	111	0	0	562 (100%)	0 (0%)	222 (100%)	0 (0%)	1	
Imken L(2009)	Morocco	Caucasian	PCR sequence	PB	135/160	133	2	0	155	5	0	268 (99.26%)	2 (0.74%)	315 (98.44%)	5 (1.56%)	0.841	

PCR sequence, polymerase chain reaction and sequencing; PCR-RFLP, polymerase chain reaction-restriction fragment length polymorphism; Others, MassARRAY or PCR-RFLP and PCR sequence

MIX, complete region

HP, hospital population; PB, population based

P_{HWE}, Value of Hardy-Weinberg equilibrium in the control group

MAF, Minimum allele frequency

Supplementary Table S3. Main characteristics of all studies on the genotype of rs2070923 included in the meta-analysis

First author (year)	Country	Ethnicity	Method	Control source	Cases/ Controls	Case			Control			Case		Control		P_{HWE}	MAF
						AA	AC	CC	AA	AC	CC	A (%)	C (%)	A (%)	C (%)		
He XJ(2012)	China	Asian	MassARRAY	HP	306/376	162	113	31	204	146	26	437 (71.41%)	175 (28.59%)	554 (73.67%)	198 (26.33%)	0.986	0.717
Grassetti D(2012)	Italy	Caucasian	PCR sequence	HP	110/53	14	54	42	5	25	23	82 (37.27%)	138 (62.73%)	35 (33.02%)	71 (66.98%)	0.629	
Tuttelmann F(2010)	Germany	Caucasian	PCR sequence	PB	159/73	26	55	78	9	26	38	107 (33.65%)	211 (66.35%)	44 (30.14%)	102 (69.86%)	0.188	
Aoki VW(2006)	America	Caucasian	PCR sequence	HP	192/96	27	72	93	12	44	40	126 (32.81%	258 (67.19%)	68 (35.42%)	124 (64.58%)	0.985	
Tanaka H(2003)	Japan	Asian	PCR sequence	PB	226/270	125	82	19	127	118	25	332 (73.45%)	120 (26.55%)	372 (68.89%)	168 (31.11%)	0.748	
Venkatesh S(2011)	India	Asian	PCR sequence	PB	100/100	25	20	55	40	0	60	70 (35.00%)	130 (65.00%)	80 (40.00%)	120 (60.00%)	0	
Jodar M(2010a)	Spain	Caucasian	PCR sequence	HP	111/50	8	39	64	5	23	22	55 (24.77%)	167 (75.23%)	33 (33.00%)	67 (67.00%)	0.776	

PCR sequence, polymerase chain reaction and sequencing

HP, hospital population; PB, population based

P_{HWE}, Value of Hardy-Weinberg equilibrium in the control group

MAF, Minimum allele frequency
